# A Serpin Released by an Entomopathogen Impairs Clot Formation in Insect Defense System

**DOI:** 10.1371/journal.pone.0069161

**Published:** 2013-07-16

**Authors:** Duarte Toubarro, Mónica M. Avila, YouJin Hao, Natesan Balasubramanian, Yingjun Jing, Rafael Montiel, Tiago Q. Faria, Rui M. Brito, Nelson Simões

**Affiliations:** 1 Centro Investigação Recursos Naturais do Centro de Biotecnologia dos Açores, Associate Laboratory of Institute for Biotechnology and Bioengineering, Department of Biology, University of Azores, Ponta Delgada, Portugal; 2 Laboratorio Nacional de Genómica para la Biodiversidad, Centro de Investigación y de Estudios Avanzados del Instituto Politécnico Nacional, Irapuato, Guanajuato, Mexico; 3 Center for Neuroscience and Cell Biology, University of Coimbra, Coimbra, Portugal; The Chinese University of Hong Kong, China

## Abstract

*Steinernema carpocapsae* is an entomopathogenic nematode widely used for the control of insect pests due to its virulence, which is mainly attributed to the ability the parasitic stage has to overcome insect defences. To identify the mechanisms underlying such a characteristic, we studied a novel serpin-like inhibitor (*sc-srp-6*) that was detected in a transcriptome analysis. Recombinant Sc-SRP-6 produced in *Escherichia coli* had a native fold of serpins belonging to the α-1-peptidase family and exhibited inhibitory activity against trypsin and α-chymotrypsin with *Ki* of 0.42×10^−7 ^M and 1.22×10^−7^ M, respectively. Functional analysis revealed that Sc-SRP-6 inhibits insect digestive enzymes, thus preventing the hydrolysis of ingested particles. Moreover, Sc-SRP-6 impaired the formation of hard clots at the injury site, a major insect defence mechanism against invasive pathogens. Sc-SRP-6 does not prevent the formation of clot fibres and the activation of prophenoloxidases but impairs the incorporation of the melanin into the clot. Binding assays showed a complex formation between Sc-SRP-6 and three proteins in the hemolymph of lepidopteran required for clotting, apolipophorin, hexamerin and trypsin-like, although the catalytic inhibition occurred exclusively in trypsin-like. This data allowed the conclusion that Sc-SRP-6 promotes nematode virulence by inhibiting insect gut juices and by impairing immune clot reaction.

## Introduction

*Steinernema carpocapsae* is an entomopathogenic nematode (EPN) in symbiosis with the bacteria *Xenorhabdus nematophila*, forming a complex highly virulent to insects, and thus, it is used globally for the biological control of pests [1]. The nematode infective juvenile (IJ) is a free-dwelling, third-stage nematode that carries bacteria in a diverticulum [2]. The IJ of steinernematids contaminates insects mostly through the mouth or anus, invading the mid-gut a few hours after exposition [3] to become established in the hemocoel where the bacteria is released and the nematode resumes its life cycle [4]. Insect death occurs approximately 48 hrs post contamination due to toxicogenesis caused by toxins and enzymes expressed by the bacteria [5], [6] and toxic factors excreted by nematodes [7], [8]. It has been reported in the literature that insect parasitic and pathogenic nematodes are able to overcome insect defences [9]–[10], which is attributed to cuticle composition and released immune suppressor molecules in steinernematids [11]. Despite the fact that effective insect defence responses against EPN have seldom been reported [12], [13], recent studies have shown that *Heterorhabditis nematophila* activates transglutaminase in *Drosophila melanogaster*, thus confirming that EPN are recognised as non-self [14] and implying the active destruction of host defence effectors by infective nematodes.

Insect defences that prevent pathogens from entering the hemocoel include the immediate sealing of wound sites by clotting and melanisation [15]. Clotting appears to serve at least three functions: to entrap and sequester invasive microorganisms, thus preventing infection; to form a physical barrier to prevent the entry of pathogens; and to facilitate wound sealing [16]. Typically, clots comprise fibres formed by humoral and cellular defence proteins and melanin resulting from the conversion of prophenoloxidase (PPO) into active phenoloxidase (PO). The activation of humoral proteins and PPO reflects an imbalance between serine proteases and their inhibitors. Several serine proteases are known to activate clotting factors in insects [17], and at least three serine proteases regulated by serpins are involved in PPO activation [18]. Although the activation of clotting factors and PPO are independent processes [19], the formation of a hard clot capable of trapping microbes and facilitating healing requires the activity of cross-linking enzymes, such as transglutaminase, and melanisation [20], [21].

Parasitic nematodes are known to express modulators of host defence reactions, which are released in excreted secreted products (ESPs) [22]. In an attempt to understand the interaction between *S. carpocapsae* and insects, an expressed sequence tag (EST) library was created and analysed, resulting in the identification of a serine protease inhibitor [23]. This inhibitor was later detected in ESPs produced by parasitic stage nematodes, suggesting its involvement in the parasitic process. In vertebrates, protease inhibitors are produced by enteric nematodes to counteract host digestive enzymes [24], [25]. Furthermore protease inhibitors released in nematode ESPs were shown to be involved in the modulation of host defences at the intestinal barrier, which is critical for the success of enteric nematodes [26]–[27].

Here, we show that *S. carpocapsae* produces a serine protease inhibitor belonging to the serpin family, which is predominantly expressed by nematodes at the parasitic stage in the insect mid-gut. The inhibitor does not affect the formation of clot fibres or the activation of PPO but impairs the formation of hard clots by blocking the interaction between clot fibres and melanin, which is likely due to the formation of complexes with clot effectors molecules.

## Materials and Methods

### Nematode and Insects

Infective juveniles of the *Steinernema carpocapsae* Breton strain were produced in larvae from the *Galleria mellonella* moth, harvested in a White trap and stored in tap water at 10°C for 1–2 months before use. Larvae from *G. mellonella*, *Pseudalaetia unipuncta* and *Spodoptera littoralis* were supplied by the insectarium in our Department.

### RNA Extraction and Full-length cDNA Cloning

The full-length *sc-srp-6* cDNA was cloned using a previously isolated 866-bp expressed sequence tag (EST) from the parasitic stage of *S. carpocapsae* (GenBank accession number GR977748.1). Total RNA was isolated as previously described [23], and first-strand cDNA was synthesised using Superscript III reverse transcriptase (Invitrogen) and an oligo (dT) primer. The full-length cDNA was produced by rapid amplification of cDNA ends (RACE) using the SMART RACE cDNA Amplification Kit (Clontech-Takara) with the SRP-5′ primer (5′-TAG AAG CGA CCG TCT TGG GTG-3′) to isolate the 5′ end and an oligo-d(T)_20_ and SRP-3′ primer (5′-TTC CCG AAG AGT GAG ACC-3′) to isolate the 3′ end. The reaction mixture contained 50 ng of cDNA, 2.5 µl 10×PCR buffer, 10 mM dNTPs, 0.4 µM of each primer, 1.6 mM MgCl_2_ and 0.2 µl *Taq* DNA polymerase in a final volume of 25 µl. The amplification comprised an initial denaturation step at 94°C for 3 min, followed by 30 cycles at 94°C for 30 s, 55°C for 30 s and 72°C for 30 s, with a final extension at 72°C for 3 min. Amplified cDNA fragments were TA-cloned into the pCR4-TOPO vector using the TOPO TA Cloning kit (Invitrogen) and send to sequence by a costumer service (STABvida, Portugal).

### *In silico* Analysis of *sc-srp-6*

The *sc-srp-6* full-length cDNA was used in a BLAST search query (http://ncbi.nlm.nih.gov/blast), and sequence alignments were created with the ClustalW program (http://www.ebi.ac.uk/clustalw). Protein motifs were predicted using SMART (http://smart.emblheidelberg.de), and SignalP 3.0 was used to identify the signal peptide (http://www.cbs.dtu.dk/services/SignalP). For phylogenetic reconstruction, sequences were aligned with Muscle (www.ebi.ac.uk/Tools/msa/muscle/). A phylogenetic tree was reconstructed with maximum likelihood using the PhyML program (www.atgc-montpellier.fr/phyml/), and robustness was assessed by the bootstrap method (100 pseudoreplicates). Human serpin was used as an out-group to root the phylogeny. A 3D structure prediction was obtained using the I-TASSER online platform (http://zhanglab.ccmb.med.umich.edu/I-TASSER).

### Analysis of Gene Expression

*Sc-srp-6* mRNA levels were profiled in different nematode stages by quantitative RT-PCR. Nematodes were produced in *G. mellonella* larvae exposed to IJ in Petri dishes padded with wet filter paper and collected from parasitised insects after dissection. L3 nematodes were isolated from the gut and hemocoel, L4 nematodes were isolated from the hemocoel and adults and L1/L2 stages were isolated from the body tissues. Approximately 20 specimens were collected from each stage from three separate exposures and homogenised in liquid nitrogen to produce cDNA as previously described. Controls without reverse transcriptase were included for each sample, and data were normalised against 18*S* rRNA. *Sc-srp-6* was amplified with SRP-6F (5′-GGG GAA GAC GAG TCAG GAG A-3′) and SRP-6R (5′-CTC GGC TTG AGG GTT GCT GA-3′) primers. The 18*S* rDNA was amplified with the 18*S*F (5′-GCT AAT CGG AAA CGA AAG TC-3′) and 18*S*R (5′-CAT CCA CCG AAT CAA GAA AG-3′) primers using an ABI 7900 HT Real Time PCR thermocycler (Applied Biosystems). Amplifications were performed in triplicate with each reaction consisting of an initial heating step at 95°C for 10 min, followed by 60 cycles of 95°C for 15 s and 60°C for 60 s. RT-PCR data were analysed using the comparative C_T_ method according to the manufacturer′s recommendations. The data are expressed as the mean ± standard error from six independent experiments. Statistical analysis was performed using a one-way ANOVA followed by Bonferroni multiple comparison tests (Statistical Package for the Social Sciences software, SPSS version 13.0). Differences were considered significant at P<0.05.

### Expression Vector Construction

The full-length *sc-srp-6* coding sequence was amplified using the forward primer SRP-6heF, which includes a *Bam*HI restriction site (5′-GGA TCC ATG TTG CCG ACT CCT AAG ACC AAT-3′) and the reverse primer SRP-6heR, which includes an *Xho*I restriction site (5′-CTC GAG ATA GAA GTC CCA CGA AGA G-3′). The 1.3-kb product was purified from a 1.5% agarose gel using the QIAEX II Gel Extraction Kit (Qiagen) and digested with *Bam*HI and *Xho*I. The fragment was then inserted into the pET23a expression vector in frame with an N-terminal T7 tag (Novagen). The vector was transformed by thermal shock into the *E. coli* C41(DE3) and Rosetta™ II(DE3) strains. The identity and integrity of the inserts were confirmed by sequencing.

### Expression of Recombinant Sc-SRP-6

A single colony from each transformed *E. coli* strain was incubated in 5 ml Luria broth (LB) containing 100 µg/ml ampicillin at 37°C with shaking at 250 rpm overnight. These cultures were used to inoculate 20 ml of LB plus antibiotics as described above and were incubated at 37°C until an OD_600_ of 0.6 was reached. Sc-SRP-6 expression was induced in both strains under several different conditions including 0.5 mM isopropyl β-D-1-thiogalactopyranoside (IPTG) at 37°C for 3 hrs, 1 mM IPTG at 37°C for 3 hrs, 0.5 mM IPTG at 20°C overnight and 1 mM IPTG at 20°C overnight. Cells from the different cultures were harvested by centrifugation at 4,000×*g* for 15 min. The pellet was then suspended in 2 ml 0.05 M Tris-HCl (pH 7.4) containing 0.5 M NaCl and 1 mg/ml lysozyme and frozen at −20°C overnight. After thawing, the lysate was supplemented with 1 ml of 1 M MgCl_2_ and 600 µl of 1 mg/ml DNAase and then incubated for 30 min at room temperature with orbital agitation. Cell debris was removed by centrifugation at 12,000×*g* for 20 min, and the supernatant was collected and analysed to detect the recombinant protein.

### Solubilisation, Refolding and Purification of Recombinant Sc-SRP-6

*E. coli* Rosetta (DE3) cells were cultured in 2 L LB, and recombinant protein expression was induced with 0.5 mM IPTG followed by 3 hrs of incubation at 37°C. Cells were recovered and lysed as described above. The lysate was washed for 4 hrs in 1 L 0.05 M Tris-HCl (pH 7.5) containing 0.5 M NaCl (TN), followed by 4 hrs in TN containing 1% Triton X-100, and the pellet with inclusion bodies was recovered by centrifugation at 6,500×*g* for 20 min at 4°C. The inclusion bodies were solubilised overnight in 40 ml of 8 M urea containing 0.7% β-mercaptoethanol under orbital agitation. The solution was adjusted to 200 ml with the same buffer and dialysed in a cellulose membrane tube against 20 mM Tris-HCl (pH 8.0) for 48 h at 4°C followed by two more buffer changes.

Solubilised proteins were concentrated using a 5000 molecular-weight cutoff membrane (Centricon, Millipore), diluted 1∶1 in 20 mM Tris (pH 8.0), 5 mM imidazole, 150 mM NaCl, 5 mM DTT and purified by nickel-chelate affinity chromatography (Ni-Sepharose, GE Healthcare) in an AKTA FPLC system (GE Healthcare). The Sc-SRP-6 fraction was concentrated and dialysed using a Centricon column and was subsequently refolded for 3 hrs in 300 mM L-arginine, 50 mM Tris (pH 8.0), 50 mM NaCl and 5 mM DTT. The refolded Sc-SRP-6 was then applied to a MonoQ column (GE Healthcare) that was equilibrated with 20 mM Tris-HCl (pH 8) and eluted in a linear gradient of 1 M NaCl to eliminate minor contaminants. The protein concentration was determined using the BCA Kit (Thermo Scientific), and purity was assessed by 10% SDS-PAGE.

### SDS-PAGE and Western Blot

Recombinant Sc-SRP-6 was analysed by 10% SDS-PAGE with a Mini-PROTEAN II gel system (Bio-Rad), and proteins were stained with Coomassie brilliant blue dye. For western blot, proteins separated by SDS-PAGE were electroblotted onto a PVDF membrane using a mini Trans-Blot Cell (Bio-Rad). The membrane was blocked with 0.01 M Tris-HCl (pH 7.5) containing 0.1 M NaCl, 5% (w/v) BSA and 0.05% (v/v) Tween-20 for 30 min at room temperature (RT) and then incubated with peroxidase-conjugated mouse anti-His_6_-tag antibody diluted (1∶5,000) in blocking solution for 2 hrs at RT. Membranes were washed three times for 10 min with 0.01 M Tris-HCl (pH 7.5), 0.1 M NaCl. Antibodies were detected by incubation with tetramethylbenzidine peroxidase substrate (Sigma) according to the manufacturer′s recommendations.

### MALDI-TOF/TOF (Matrix-assisted Laser Desorption/Ionization-Time-of-Flight) Tandem Mass Spectrometry

Proteins were digested in-gel with trypsin, and the peptides were purified and concentrated using an R2 pore microcolumn. The peptides were applied to a MALDI plate with 0.5 µl α-cyano-4-hydroxycinnamic acid matrix (5 mg/µl in 50% acetonitrile, 5% formic acid) and prepared for MALDI as previously described [28]. The *m/z* spectra were acquired in a 4,700 Proteomics Analyzer MALDI-TOF/TOF (Applied Biosystems) in the MS and MS/MS modes. Searches were performed with a minimum mass accuracy of 50 ppm for the parent ions, an error of 0.3 Da for the fragments, one missed cleavage in the peptide masses and carbamidomethylation of Cys and oxidation of Met as fixed and variable amino acid modifications, respectively. The confidence threshold for protein identification was set to 95% (p<0.05). To obtain the highest confidence score for the identification of proteins involved in serpin complexes, the masses originating from Sc-SRP-6 were excluded from the peptide mass list used in the database search. Protein identification was performed using the MASCOT program (www.matrixscience.com) to search the UniProtKB database (downloaded on 10/06/2012).

### Circular Dichroism of Recombinant Sc-SRP-6

To assess the correct folding of recombinant Sc-SRP-6, the far-UV circular dichroism (CD) spectrum of the protein was acquired. Purified protein was run on size-exclusion Sephadex 200 chromatography columns to remove putative aggregates and eluted in 50 mM phosphate buffer (PB) (pH 8.0). CD experiments were performed on a DSM-20 CD spectrophotopolarimeter (OLIS) controlled by GlobalWorks software using protein concentrations ranging from 0.15 to 0.35 mg/ml. Far-UV CD spectra were recorded between 190 and 260 nm using a 1-mm path length cuvette. CD spectra were run with a step-resolution of 1 nm, an integration time of 5 sec, and slit width of 0.6 nm, at 37°C. The spectra were averaged over two scans and corrected by subtraction of the buffer signal. Data are expressed as the mean residue molar ellipticity [Θ]MRW, which is defined as [Θ]MRW = Θobs (0.1MRW)/(lc), where Θobs is the observed ellipticity in millidegrees, MRW is the mean residue weight, c is the concentration in milligrams per millilitre and l is the cuvette path length in centimetres. The secondary structure contents were calculated with the CONTINL, CDSSTR and Selcon3 algorithms [29]–[31] against the CLSTR reference basis set, which contains soluble and denatured proteins with known secondary structure.

### Sc-SRP-6 Specificity

The inhibitory activity of purified Sc-SRP-6 was tested against trypsin, α-chymotrypsin and elastase from bovine pancreas, subtilisin from *Bacillus licheniformis* and thrombin from bovine plasma. Twenty microlitres of Sc-SRP-6 (3.5 µg/µl) was incubated with 10 µl of each enzyme (1 µg/µl) for 10 min at 37°C in microtiter plates with the total volume adjusted to 45 µl with 0.1 M Tris-HCl (pH 8) containing 0.1 M NaCl and 1 mM CaCl_2_. The hydrolytic activity of the remaining enzyme was quantified in a final concentration of 1 mM of the BApNA, Suc-AAPFpNA, Suc-AAPLpNA, Z-GGLpNA and BPVA-pNA (Sigma-Aldrich) chromogenic substrates for trypsin, α-chymotrypsin, elastase, subtilisin and thrombin (Sigma-Aldrich), respectively. Sc-SRP-6 was replaced with buffer in the control reactions. The formation of p-nitroaniline was monitored in an ELISA microplate reader at 405 nm, and the percentage of enzyme inhibition caused by Sc-SRP-6 was calculated as follows: % inhibition = ((total enzyme activity units – residual enzyme activity units)/total enzyme activity units) * 100.

### Kinetic of Sc-SRP-6

The activity of trypsin or chymotrypsin was determined with specific substrates (1 mM) in the presence of different concentration of Sc-SRP-6 (0, 8, 22 and 30 µM) under the conditions described above. The fractional activity (velocity of enzyme with Sc-SRP-6/velocity of enzyme without Sc-SRP-6) was plotted against the ratio of serpin/enzyme concentrations, and the *x*-axis intercept as a value for the stoichiometry of inhibition (SI) was determined by linear regression analysis. The inhibition constant (*Ki*) for Sc-SRP-6 against trypsin and chymotrypsin was estimated using a Dixon plot. A single regression line for each substrate concentration (0.05, 1 and 2 mM) was obtained, and the *Ki* was calculated from the intersection of the three lines. The inhibitory mechanism was determined using Lineweaver-Burk plots. Regression lines for trypsin and chymotrypsin were produced using the inverse of the initial rate for each Sc-SRP-6 concentration plotted against the inverse of the substrate concentration.

To determine the optimal pH, Sc-SRP-6 was mixed with each enzyme, and the pH adjusted to 5, 6 and 7 with 0.1 M PB, 0.1 M NaCl and to 8 and 9 with 0.1 M Tris-HCl, 0.1 M NaCl. After incubation for 10 min at 37°C, the inhibition of enzyme activity was determined under the conditions described above.

### Formation of SDS-stable Complexes

To identify serpin/trypsin and serpin/α-chymotrypsin complexes, reactions between serpin and enzyme concentrations equal to and above the SI were analysed by SDS-PAGE, under non-reducing conditions. To search for serpin and plasma protein SDS-stable complexes, Sc-SRP-6 was incubated with LPS-activated plasma (prepared as described in the phenoloxidase activation section) at 1∶20 and 1∶10 concentration ratios in 50 mM Tris-HCl (pH 8.0) for 30 min at 37°C. The reaction mixture was boiled at 90°C in non-reducing loading buffer for 10 min and then resolved on a 10% SDS-PAGE gel. In controls with phenylmethylsulfonyl fluoride (PMSF) the samples were preincubated with 5 mM PMSF for 10 min at RT. Sc-SRP-6 and complexes were detected in-gel using the InVision™ His-tag Stain signal (Novex) and excised after staining with Coomassie brilliant blue.

### Isolation of Insect Mid-gut Proteases and Inhibition Assays

Digestive enzymes were extracted from chilled and excised mid-guts from 20 fourth-instar *G. mellonella*, *P. unipuncta* and *S. littoralis* larvae. After rinsing in ice-cold 20 mM Tris-HCl (pH 8), the mid-guts were homogenised on ice in 1 ml of buffer using a Polytron homogeniser at 12,000 rpm. Debris was removed by centrifugation at 15,000×*g* for 20 min at 4°C, and the supernatant was filtered through a 0.2 µm nitrocellulose membrane (Millipore). The filtrate was fractionated in a Sephacryl S-200 column (GE) in 20 mM Tris-HCl (pH 8), and the fractions were screened for trypsin and chymotrypsin activities with 2 mM BApNA and Suc-AAPFpNA, respectively. Active fractions for both enzymes were pooled. In the inhibition assays, insect enzymes were pre-incubated with 30 µM Sc-SRP-6 in 20 mM Tris-HCl (pH 8) for 30 min at 30°C. The activity was determined using the referred specific substrates for each protease. Assays were performed in triplicate.

### Insect Feeding Assays

*P. unipuncta* neonates were fed an artificial diet supplemented with 200 µM Sc-SRP-6 at 0.2% (w/v), which was replaced under the same conditions every 2 days. In the controls, Sc-SRP-6 was replaced with Ringer solution. To follow the digestion of ingested materials, albumin-bromphenol blue was prepared as described [32] and added to the artificial diet. Twenty larvae were individually dispersed in 24-well multi-well plates with perforated lids and maintained at 25°C with a 16/8 hrs (L/D) cycle. The assay was performed in triplicate. Larval weight and mortality were recorded every 2 days and compared using one-way ANOVA. Differences were considered significant at P<0.05.

### Phenoloxidase Activation Analysis

PO activation in *G. mellonella* plasma was quantified as previously described [21]. The hemolymph was collected by puncturing the ventral pro-leg of fourth-instar larvae and draining it into an anticoagulant solution (Ringer containing 20 mM EDTA instead of CaCl_2_) at a 5∶1 final ratio (v/v). The solution was centrifuged at 1,500×*g* for 3 min at 4°C to remove the hemocytes, and 40 µl of the plasma solution was added to 10 µl of Sc-SRP-6 (3 µM/µl) and incubated for 5 min on ice. As a control, Sc-SRP-6 was replaced with the anticoagulant. The samples were then activated with 5.5 µl (10 µg/ml) of lipopolysaccharide (LPS *E. coli* O55:B5, Sigma) dissolved in 50 mM Tris-HCl (pH 7.5), 0.1 M NaCl and incubated for 15 min at 25°C. The plasma produced was centrifuged at 5,000×*g* for 5 min at room temperature, and the supernatant was recovered and supplemented with 20 µl of 15 mM L-DOPA (Sigma), dissolved in 20 mM Tris-HCl (pH 7.5) and incubated for 15 min at 37°C. The reaction was monitored at 490 nm using an ELISA microplate reader (Bio-Rad). PO activation was measured as the relative change in OD at reaction time points 0 and 15 min. Experiments were performed in triplicate.

### Analysis of Clot Formation

A drop clot assay was performed by adding 30 µM Sc-SRP-6 to a plasma drop on a slide that was then activated with LPS (10 µg/ml). The slide was placed in a humidified chamber for 30 min. To measure the incorporation of melanin in clots after incubation with and without Sc-SRP-6, the supernatant and adherent materials were carefully separated by aspiration with a fine tip followed by visualisation and photography by light microscopy. To determine whether clot fibres formed, plasma drops prepared and incubated as above were fixed with 2% glutaraldehyde in 10 mM phosphate buffer (PB) (pH 7) at 4°C for 15 min. The glutaraldehyde was removed from the slides by carefully washing with 10 mM PB, and it was replaced with 70% ethanol. The floating clot strands were carefully transferred to a new slide and observed by phase-contrast microscopy. In the same assay, a portion of the clot was transferred to PB plus 8% NaCl, stained with 10 mg/ml peanut agglutinin conjugated with FITC (PNA-FITC, Sigma) and observed under a fluorescence microscope (Zeiss) at 480 nm. An encapsulation assay was performed with Sephadex G200 beads. Beads were added to the LPS-activated plasma, incubated for 30 min in a humidified chamber and fixed in 2% glutaraldehyde. Preparations were processed for scanning electron microscopy as previously described [33].

### Analysis of Reaction from Injury

Fourth-instar larvae were lightly anesthetised by chilling and then pierced with an ultrafine needle in the ventral midline between segments A3 and A4 under a stereomicroscope. Fifteen microlitres of Sc-SRP-6 (0.3 µM) was applied to the wounds, and the larvae were maintained in Petri dishes for 30 min at RT. The larvae were then dissected to expose the incision site, and the wounds analysed and photographed under a stereomicroscope.

## Results

### Sc-SRP-6 is a Serpin-like Inhibitor of the I4 Family

**Figure 1:**
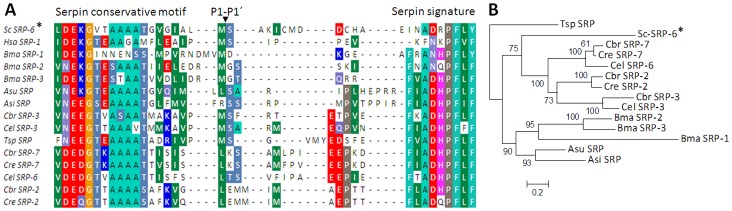
Sc-SRP-6 shares RCL signatures and has phylogenetic relationships with parasitic nematode serpins. **A:** Multiple alignment of the C-terminus of serpins from different species highlight a conserved GVTA motif (346–349), the serpin signature INADRP (373–378) and a predicted P1–P1′ cleavage site (Met360–Sep361). High variability is observed in the region immediately proximal to P1 position of the RSL. **B:** Phylogenetic tree reconstructed by Maximum Likelihood using PhyML with robustness assessed by the bootstrap method (1000 pseudoreplicates). Human serpin was used as an outgroup to root the phylogeny. Alignments were performed with the amino-acid sequences of the following serpins: *Caenorhabditis briggsae* Cbr-SRP-3 (XP_002647307); *C. elegans* Cel-SRP-3 (NP_503528); *Brugia malayi* Bml-SRP-1 (AAB65745), BML-SRP-2 (XP_001893428) and BML-SRP-3 (XP_001896647); *Trichinella sp*. Tr-SRP (ABI32311); *Ascaris suum* Asn-SRP-1 (ADY44079); and *Anisakis simplex* Asm-SRP-1 (CBX25525) and Human Hsa-SRP-1 (ABV21360).

Full-length cDNA representing a novel *S. carpocapsae* protease inhibitor (Sc-SRP-6) was isolated based on a previously identified EST and the sequence submitted to GenBank (accession number HM586104). The complete cDNA was 1,297 bp including a 1,191-bp open reading frame (ORF) and a 152-bp 3′-untranslated region with a putative polyadenylation signal (AATAAA). The ORF was predicted to encode a 397-amino acid polypeptide with a single serpin domain spanning residues 35–396, a 19-residue pre-protein and an N-terminal signal peptide spanning residues 16–17, which suggests that this protein is involved in a classical secretory mechanism (Figure S1). The mature protein was predicted to have a mass of 42 kDa and a p*I* of 4.5. BLAST analysis identified a reactive centre loop (RCL) in the C-terminal portion of the protein with specific serpin features such as the conserved serpin motif GVTA (346–349), the serpin signature INADRP (373–378) and a P1–P1′ cleavage site (M360-S361) (Figure 1A).

MEROPS analysis suggested that Sc-SRP-6 is homologous to family I4 unassigned peptidase inhibitors of the clan α-1-peptidase inhibitor. A BLAST search of the GenBank database showed that Sc-SRP-6 has a higher level of identity to nematode serpins than to those from vertebrates, with the maximum identity (30%) shared with a secreted serpin from the parasitic nematode *Trichinella spiralis*. However, at the family level, the phylogenetic tree is in agreement with the proposed phylogeny of nematodes [34], with Sc-SRP-6 in the Rhabditida group separated from Ascaridida (*i.e., Ascaris suum* and *Anisakis simplex*) and Spirurida (*B. malayi*). In the Rhabditida group, all the *Caenorhabditis* spp. serpins clustered together, whereas Sc-SRP-6 was positioned outside of this cluster in a single branch. All groupings are well supported by bootstrap values (Figure 1B).

A predicted 3D model for Sc-SRP-6 was computed using the ITASSER server, which yielded an estimated model accuracy of 0.69±0.12 (TM-score) and a confidence score of -0.15 for the top template alignment. The predicted structure demonstrated a typical serpin fold comprising 3 large β-sheets and 9 α-helices (Figure S2), which is similar to the native fold of other serpins. Highly similar structures were found between Sc-SRP-6 and *Ixodes ricinus* serpin (PDB: 3NDA) (TM-scores of 0.928), which is involved in the inhibition of host inflammation and platelet aggregation [35], and the human serpin antithrombin III (PDB: 1DZH) and plasminogen activator inhibitor (PDB: 3CVM) (TM-scores of 0.887 and 0.865), which is also involved in blood coagulation. The RCL of Sc-SRP-6 was predicted in S4 of the “B” β-strand and had the highest identity (36%) with human plasminogen activator inhibitor (1a7CA). The RCL in Sc-SRP-6 contains conserved small hydrophobic amino acids at positions P9–P12 at the base of the loop, which are thought to promote the exposure of scissile bond sites [36].

### The Invasive Nematode Stage Expressed the Highest Level of *sc-srp-6*

**Figure 2:**
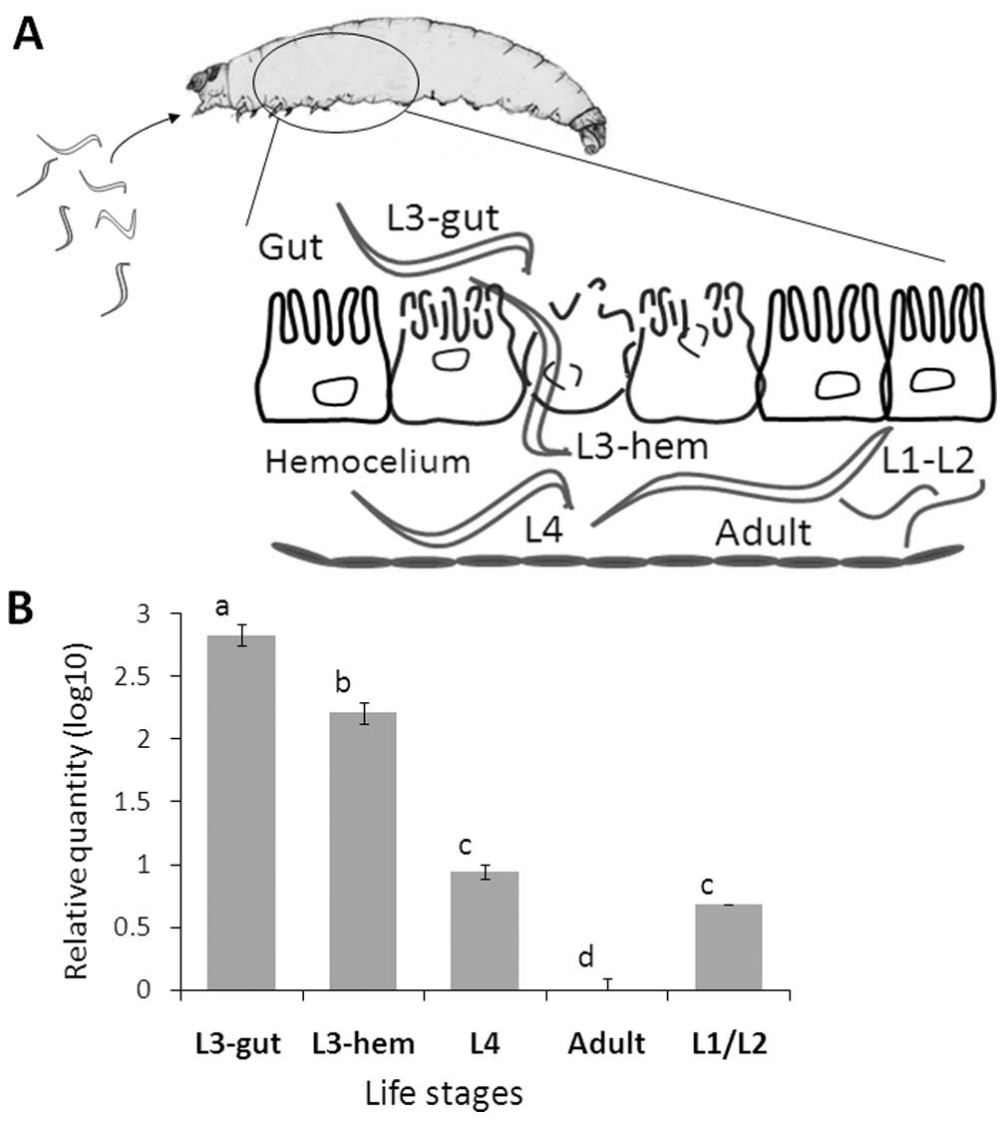
Sc-SRP-6 is upregulated in invasive parasites. **A**: The *S. carpocapsae* parasitic process. Infective juveniles (IJs) exposed to *G. mellonella* larvae recover and enter the insect mid-gut (L3-gut) to invade the intestinal barrier to become established in the hemocoel (L3-hem) where they molt (L4) and resume the life cycle in the insect carcass (adults, L1 and L2). **B:** Sc-SRP-6 relative expression was determined by real-time PCR using 18S as a control. Nematodes in each phase were collected from parasitised insects: L3-gut – third juveniles inside gut lumen; L3-hem – third juveniles in the hemocoel; L4– forth juveniles; and Adults – male and female; L1/L2– a pool of first and second juveniles. The bars represent standard deviations from three independent replicates. The different letters indicate significant differences (p<0.05).

The expression of *sc-srp-6* transcripts throughout the parasitic cycle was analysed in nematodes at different stages of development in parasitised *G. mellonella* larvae (Figure 2A). At 6 hrs post exposure (HPE), L3 collected from the digestive tract of exposed insects expressed the highest level of *sc-srp-6* (Figure 2B). L3 collected from the hemocoel at 17 HPE expressed lower levels of *sc-srp-6* than those in the gut, and L4 collected from the hemocoel at 36 HPE expressed lower levels of *sc-srp-6* than L3 in the gut or hemocoel. Adults collected from insect cadavers at 56 HPE did not express *sc-srp-6* at all. L1 and L2 harvested at 72 HPE expressed low levels of *sc-srp-6* mRNA. These data demonstrate that *sc-srp-6* is upregulated during the invasive phase before nematodes cross the mid-gut barrier.

### Production and Folding of Recombinant Sc-SRP-6

**Figure 3:**
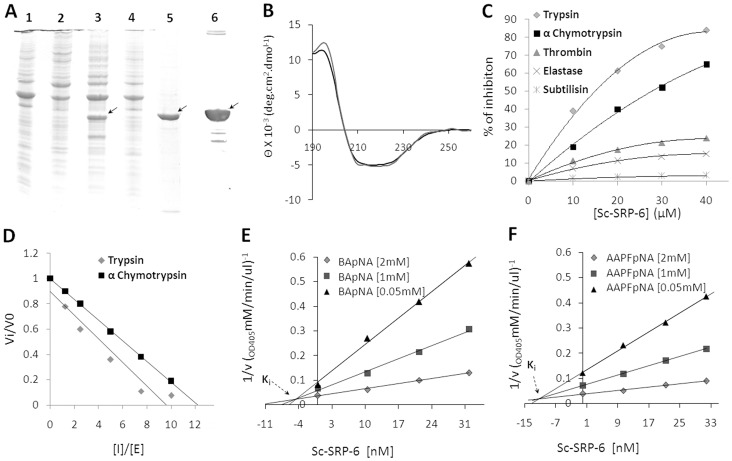
Recombinant Sc-SRP-6 inhibits chymotrypsin-like enzymes. **A:** Expression of Sc-SRP-6 after induction with 1 M IPTG at 37°C for 3 h (1), 0.5 M IPTG at 20°C overnight (2), 0.5 M IPTG at 37°C for 3 h (3), and 1 M IPTG at 20°C overnight (4). Sc-SRP-6 migrated as a single band at 42 kDa in 10% SDS-PAGE under reducing conditions (5). The identity of Sc-SRP-6 was confirmed by western blot using an anti-His_6_-tag antibody (6). **B:** Far-UV CD spectra of Sc-SRP-6. The spectra was obtained at 22°C (discontinue line) and 37°C (line). **C: S**pecificity of recombinant Sc-SRP-6 for different serine proteases. Sc-SRP-6 was incubated with enzymes at different molar ratios for 10 min at 37°C, and residual enzymatic activity was measured using specific substrates. **D:** Stoichiometry of the inhibition of Sc-SRP-6 was determined against trypsin and α-chymotrypsin. **E** and **F:** Ki of Sc-SRP-6 for trypsin and α-chymotrypsin (0.42×10^−7^ M and 1.22×10^−7^ M). The Ki was graphically determined by Dixon plots using different concentrations of trypsin and α-chymotrypsin specific substrates (BApNA and Suc-AAPFpNA).

Recombinant Sc-SRP-6 was expressed in insoluble inclusion bodies in both of the *E. coli* strains used and under all of the tested induction conditions. Optimal production was achieved in Rosetta™ 2(DE3) cells induced with 0.5 mM IPTG for 3 hrs at 37°C (Figure 3A). The folding and purification of recombinant Sc-SRP-6 was achieved as described in the material and methods. Recombinant Sc-SRP-6 migrated under reducing conditions as a single 42 kDa band on a 10% SDS-PAGE, which is close to the predicted molecular mass. Recombinant Sc-SRP-6 was confirmed by western blot using an anti-His_6_-tag antibody, and its identify was validated by MALDI-in Ms and MS/MS, which resulted in the successful identification of 13 peptides matching the predicted Sc-SRP-6 sequence, corresponding to 54% sequence coverage with a significance score of 462 (P<0.05) (Table S1). The recombinant Sc-SRP-6-fold was analysed by recording the CD spectrum. The far-UV CD spectra presented a broad negative region in the 208–222 nm range with a minimum peak at 222 nm proceeded by a shoulder at 208 nm (Figure 3B), which is highly similar to that reported for canonical serpins [37]. Based on a deconvoluted spectrum, it was calculated that the Sc-SRP-6 secondary structure contains 15–20% α-helices, 30–35% β-strands, 20–25% β-turns and 23% random coils, which is in agreement with values (22% α-helical and 35% β-strand) predicted by I-TASSER.

### Sc-SRP-6 is an Inhibitor of Chymotrypsin-like Enzymes

**Figure 4:**
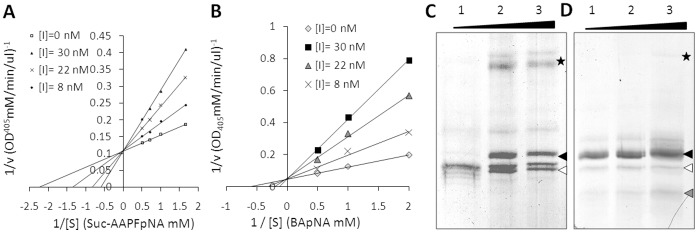
Sc-SRP-6 inhibits chymotrypsin-like enzymes by a classical mechanism involving RCL. **A–B:** Lineweaver-Burk double reciprocal plots showing that Sc-SRP-6 is a competitive inhibitor of trypsin and α-chymotrypsin. C – D: Formation of SDS-stable complexes of Sc-SRP-6/trypsin and Sc-SRP-6/α-chymotrypsin. An increased SI for Sc-SRP-6 was used (5, 10 and 15 in wells 1, 2 and 3). A serpin/protease complex (asterisk), a serpin (black arrow), a hydrolysed serpin (white arrows) and a degraded serpin (grey arrow) are indicated at specific sites in each gel.

Sc-SRP-6 was shown to inhibit trypsin and α-chymotrypsin in a dose-dependent manner, to have residual activity in thrombin and elastase and to lack activity in subtilisin (Figure 3C). The inhibitory activity of Sc-SRP-6 against trypsin and α-chymotrypsin was not significantly different (P≤0.05) at pH 6, 7 and 8 but was significantly higher (1.5-fold, P≤0.05) at pH 9 (Figure S3). The stoichiometry of inhibition for Sc-SRP-6 was 9.6 and 12.2 for trypsin and α-chymotrypsin, respectively (Figure 3D). *K*_i_ values of 0.42×10^−7^ M and 1.22×10^−7^ M for trypsin and α-chymotrypsin were graphically determined using Dixon plots (Figure 3E–F). A competitive inhibitory mechanism involving the RCL of Sc-SRP-6 was revealed by Lineweaver-Burk double reciprocal plots (Figure 4A–B) and the formation of SDS-stable complexes with trypsin and α-chymotrypsin (Figure 4C–D).

The incubation of Sc-SRP-6 with trypsin resulted in a 70 kDa band corresponding to the enzyme/inhibitor stable complex (23.8 kDa plus 43 kDa). One band corresponded to the native serpin and another that was consistent with cleavage of the exposed RCL of serpin (approximately 5 kDa less than native serpin) was also detected. The serpin/chymotrypsin complex was detected only when an excess of inhibitor was used (20∶1), confirming that the stoichiometry value for the inhibition of α-chymotrypsin was higher than that for trypsin. One band that was consistent with Sc-SRP-6 cleavage in RCL was visible after incubation with α-chymotrypsin, whereas Sc-SRP-6 degradation bands were also visible, thus indicating the simultaneous occurrence of hydrolysis and inhibition. To confirm that the serpin/enzyme complex was formed via the enzyme active site, the enzymes were preincubated with PMSF. In the presence of PMSF the formation of serpin/chymotrypsin or serpin/trypsin complexes were completely abolished.

### Sc-SRP-6 Protects Pathogens from the Effects of Insect Digestive Enzymes

**Figure 5:**
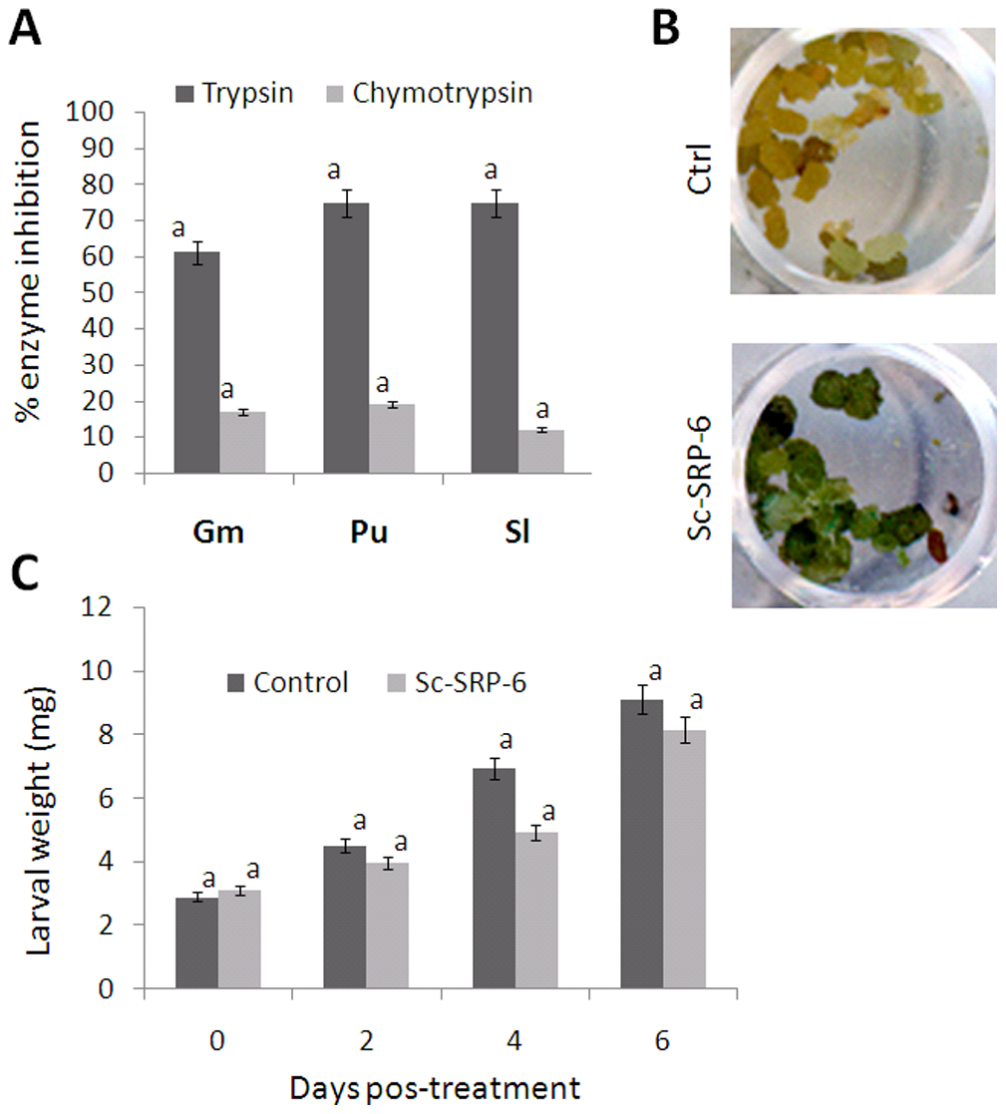
Sc-SRP-6 inhibits insect midgut enzymes. **A:** Inhibitory activity of Sc-SRP-6 against trypsin and chymotrypsin in insect digestive juices. The juices were partially fractionated by gel filtration, and the inhibitory activity tested using BApNA and AAPPpNA substrates for trypsin and chymotrypsin. **B:** *P. unipuncta* larvae fed a diet incorporated with albumin-bromphenol blue show faeces with no traces of colour (control), and larvae fed on the same diet supplemented with 0.2% Sc-SRP-6 (w/v) retained the coloured compound. **C:** Incremental weight of larvae treated with 0.2% Sc-SRP-6 (w/v) compared with untreated controls. The bars represent standard deviations from three independent replicates. The different letters indicate significant differences (p<0.05).

The activity of Sc-SRP-6 against lepidopteran digestive enzymes was tested *in vitro* and *in vivo*. Sc-SRP-6 substantially reduced the trypsin activity in insect gut juices in *in vitro* assays (Figure 4A). The inhibition of the *P. unipuncta* and *S. littoralis* trypsins (75%) was stronger than that in *G. mellonella* (63%) without significant differences among the insects (P≤0.05). The inhibition of chymotrypsin from the three insects did not exceed 20% without significant differences among them (P≤0.05). Assays conducted *in vivo* with these insects demonstrated that Sc-SRP-6 did not cause any significant modification in larval weight with respect to the control (Figure 5C). In addition, any mortality in larvae treated with Sc-SRP-6 was recorded. However, a Sc-SRP-6 effect on the digestion of ingested particles was observed. Faeces from insects fed with a diet coloured with albumin-bromphenol blue and treated with Sc-SRP-6 maintained the colour, whereas the faeces of insects fed with a diet lacking Sc-SRP-6 were uncoloured (Figure 5C). These observations suggest that Sc-SRP-6 prevents the hydrolysis of food particles and therefore is likely to protect invasive nematodes from aggressive proteolytic enzyme activity.

### Sc-SRP-6 Affects the Insect Response to Injury

**Figure 6:**
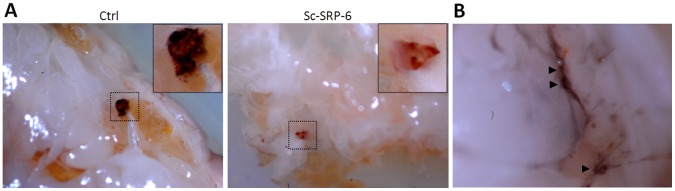
Sc-SRP-6 affects insect responses to injury. **A:** A melanised plug was identified in the wound site of untreated larvae shortly after injury (control), but no plug was formed in larvae treated with Sc-SRP-6. The larvae were punctured in the ventral midline using a 0.5 mm steel syringe in 3–4 intersegments. Control larvae were treated with 15 µl of buffer. **B:** Concavities in the treated larvae (such as the wound site) accumulates darkened hemolymph (arrowheads), providing evidence of melanisation.

We investigated the effect of Sc-SRP-6 on the response of *G. mellonella* larvae to physical injury by comparing wounded larvae treated with either Sc-SRP-6 or a control solution. In the control larvae, a plug formed at the wound site was visible shortly after injury with intense melanisation. In contrast, larvae treated with Sc-SRP-6 did not form a plug at the wound site, and there was no evidence of melanisation around the edges of the wound (Figure 6A). However, the hemolymph of treated larvae became darker following injury (this was particularly evident in concavities around the wound site) and remained dark for more than 1 hr. The dark fluid could be removed by aspiration, and no visible melanisation in the tissues was observed (Figure 6B). This assay demonstrated that Sc-SRP-6 prevents wound sealing through the formation of insoluble plugs, which are normally generated by a clot reaction and melanisation.

### Sc-SRP-6 does not Inhibit the Activation of Clot Proteins or PPO

**Figure 7:**
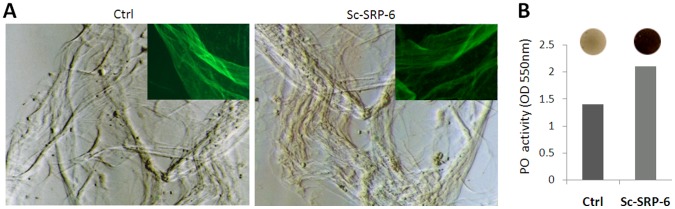
Sc-SRP-6 does not inhibit fibres formation or the activation of PPO. **A:** Clot fibres visualised by phase contrast microscopy in LPS-elicited plasma of *G. mellonella* with and without Sc-SRP-6. Framed are fibres stained with PNA-FITC and visualised by fluorescence microscopy. **B:** Sc-SRP-6 does not inhibit PO-mediated melanisation of LPS-elicited plasma but instead increased by 2-fold PO activity.

The effect of Sc-SRP-6 on clot formation and PPO activation was investigated *in vitro* using insect plasma. We initially considered the formation of protein strands in plasma, which can be elicited using bacterial LPS. In the elicited plasma, the formation of fibres was clearly observed by SEM in both the control plasma and samples treated with Sc-SRP-6. The presence of clot filaments was confirmed by labelling the fibres with PNA (Figure 7A).

The impact of Sc-SRP-6 on PPO activation was investigated in two different assays, and both showed that serpin had no significant inhibitory effect. The first assay used DOPA as a substrate and showed that PO activity was not inhibited but instead increased up to 2-fold in the presence of Sc-SRP-6 (Figure 7B). The second approach was a drop assay using *G. mellonella* plasma stimulated with LPS, which confirmed the activation of PPO and plasma melanisation in the controls and in treatments with Sc-SRP-6. These data confirm that Sc-SRP-6 does not inhibit the formation of clot fibres or the activation of PPO.

### Sc-SRP-6 Inhibits the Formation of Hardened Clots

**Figure 8:**
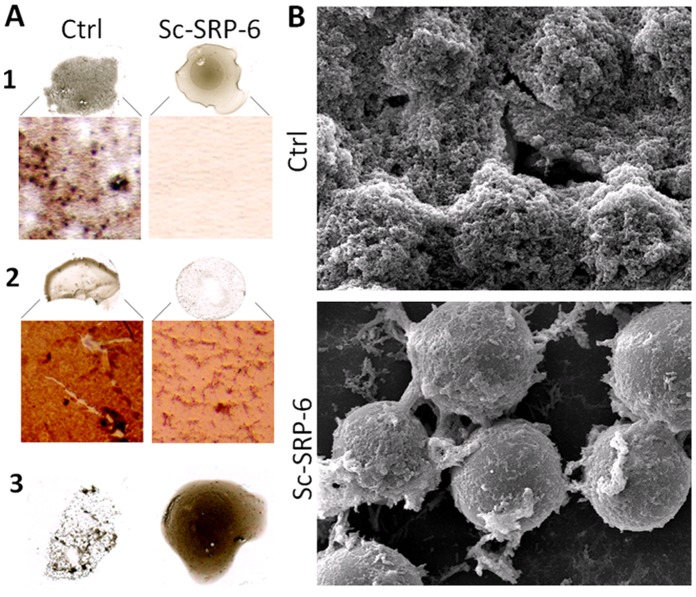
Sc-SRP-6 impairs the formation of hardened clots. **A1:** In the drop clot assay, melanin aggregates were easily recognised in controls by optical microscopy, but they were not visible in plasma treated with Sc-SRP-6 a few minutes after the plasma was stimulated. **A2:** Approximately 30 min later, material adherent to lamina treated with Sc-SRP-6 remains clear, indicating that melanin was not incorporated into the clot. **A3:** The clot supernatant formed in plasma treated with Sc-SRP-6 remains dark whereas the control supernatant is clear. **B:** Scanning electron micrographs show that beads in treated and untreated plasma were attached to clot filaments forming aggregates, but full encapsulation was inhibited in the presence of Sc-SRP-6.

Melanin aggregates in *G. mellonella* plasma were visible a few minutes after stimulation with LPS, and dark nodules were easily recognised by optical microscopy. However, neither melanin aggregates nor dark nodules were observed in plasma treated with Sc-SRP-6 (Figure 8A). After 30 min, melanin was incorporated into clots in control plasma samples, but not in plasma treated with Sc-SRP-6. Whereas a hardened and melanised clot was formed in the control plasma, the treated plasma remained dark, and there was no interaction between the clot fibres and melanin. These data indicate that Sc-SRP-6 prevents the formation of melanin nodules and their incorporation into clot strands. Therefore, we analysed the effect of Sc-SRP-6 on the encapsulation of foreign bodies using beads.

Scanning electron micrographs showed that the beads were incorporated in large aggregates in the treated and untreated plasma. However, the beads were completely enclosed by clots in the untreated plasma, whereas clot strands remained visible without the incorporation of other materials in the presence of Sc-SRP-6 (Figure 8B). These observations suggest that although the beads were captured in clot filaments, they were not encapsulated, indicating that Sc-SRP-6 inhibits the incorporation of melanin into clots and interrupts the hardening process.

### Sc-SRP-6 Formed Complexes with Insect Plasma Proteins

**Figure 9:**
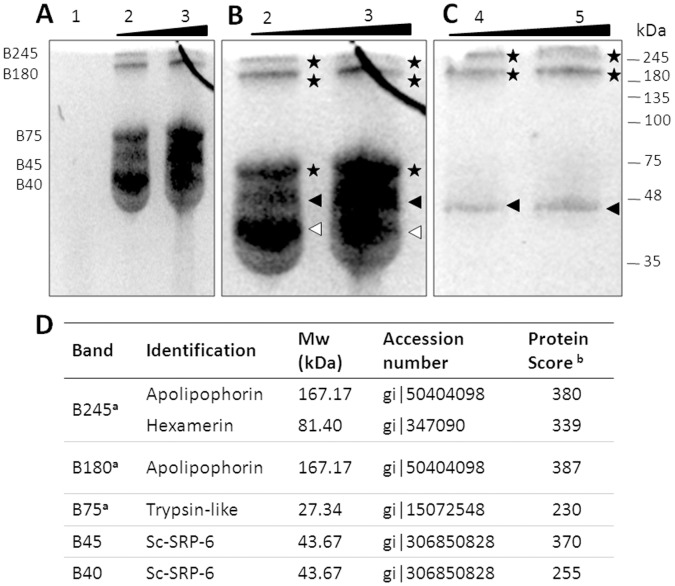
Sc-SRP-6 formed SDS-stable complexes with *G. mellonella* plasma proteins. **A and B:** Complex formation between Sc-SRP-6 and plasma proteins at serpin/plasma proteins ratios of 4 (well 1), 15 (well 2) and 14 (well 3). **C:** Plasma proteins incubated with Sc-SRP-6 in the presence of 1.0 mM PMSF. After incubation for 30 min at 37°C, reactions were heated to 90°C under non-reducing conditions for 10 min and resolved in a 10% SDS-PAGE gel. The mobility of the serpin-protease complex (asterisk), serpin (black arrow) and hydrolysed serpin (white arrows) are indicated on the side of each gel. **D:** Immune-detected bands were analysed by MALDI-TOF/TOF. (a): For serpin complexes, masses originating from Sc-SRP-6 were excluded from the peptide mass list used for database searching to ensure the highest confidence scores for identified plasma proteins. (b): The protein score probability limit (where P<0.05) was 86.

To address the Sc-SRP-6 targets in plasma proteins, we assayed for the formation of stable complexes. The exposure of Sc-SRP-6 to LPS-activated plasma led to the visualisation of five bands in which Sc-SRP-6 was immune-detected (Figures 9 A-B). Using MS/MS, the bands at approximately 45 kDa and another below 45 kDa were identified as Sc-SRP-6, and the molecular weight of the smallest band was consistent with cleaved serpin (Figure 9 D). Three other bands at 75, 180 and 245 kDa were shown to correspond to Sc-SRP-6 complexes (Table S2).

The 75 kDa band was identified as a complex of Sc-SRP-6 and a trypsin-like protein from *G. mellonella*. The approximately 180 kDa complex was identified as a mix of Sc-SRP-6 and apolipophorin from *G. mellonella*, which is consistent with the size of each protein (45 and 167 kDa). The approximately 245 kDa complex was identified as a mixture of two *G. mellonella* proteins, apolipophorin and hexamerin, and Sc-SRP-6, which is also in accordance with the sum of the molecular weights (167, 81 and 45 kDa). Catalytic complexes were investigated by competition with the inhibitor PMSF. The formation of the 75 kDa complex was inhibited, whereas the 245 and 180 kDa complexes were not (Figure 9 B-C). These data indicate that the Sc-SRP-6/plasma trypsin complex was formed by a covalent bound between the protease active site and the serpin RCL.

## Discussion

We have characterised a novel serine protease inhibitor (Sc-SRP-6) that is upregulated in the parasitic larvae of *S. carpocapsae,* an entomopathogenic nematode that uses an unknown mechanism to overcome host defences.

Sc-SRP-6 belongs to the I4 serpin family of peptidase inhibitors based on the presence of a conserved serpin signature [38] despite extensive variability in the scissile bond sequence that was observed within the active-site loop (particularly near the P1 position) compared with homologous serpins. This variability is thought to affect substrate selectivity, particularly in the context of adaptive properties resulting from the evolutionary arms race between hosts and parasites [24].

Biochemical analyses with recombinant Sc-SRP-6 showed that it efficiently inhibits chymotrypsin-like enzymes by forming a canonical enzyme/serpin stable complex with trypsin and α-chymotrypsin. *In silico* and CD data suggested that recombinant Sc-SRP-6 has an identical fold as the mouse serpin trypsin inhibitor with a composition consisting of 24–29% α-helices, 31% β-sheets, and 10–11% β-turns [37] and of plasminogen activator inhibitor-1, which is composed of 29% α-helices, 19% β-strands, 24% β-turns, and 27% random coils [39] and differs from that of human α-1-antitrypsin, which is composed of 48% α-helices, 25% β-sheets, and 9% β-turns [37].

Serpins released by intestinal parasitic nematodes of vertebrates are thought to neutralise host digestive enzymes. Our data showed that Sc-SRP-6 inhibits most of the trypsin-like activity in assays using lepidopteran gut juice, which predominantly contains trypsin. Furthermore, food particles containing Sc-SRP-6 were subject to less hydrolysis than control particles. Therefore, we hypothesise that Sc-SRP-6 protects parasites during their passage through the insect gut as proposed for the intestinal nematodes *A. simplex* and *A. ceylanicum* [24].

Bearing in mind the homology of Sc-SRP-6 with serpins participating in vertebrate coagulation pathways, which are analogous to the coagulation pathways used as a primary defence mechanism in arthropods [40], we analysed the effect of these inhibitors in insect clotting. This analysis showed that Sc-SRP-6 impairs the formation of hard clots in response to exogenous stimulants, allowing foreign bodies to aggregate but not become encased in a hard, melanised clot.

It is well known that the encapsulation process requires clot formation and PPO activation, both involving enzymatic cascades regulated by an imbalance in serine proteases and serpins [41]. First of all we hypothesised that Sc-SRP-6 inhibited one or more of these processes, but this hypothesis was proven wrong when we performed an *in vitro* drop assay. Although clot protein strands were formed and PPO was activated in the presence of Sc-SRP-6 (resulting in the formation of melanin and thus the visible darkening of the plasma), the clots were soft and non-melanised. This observation suggested that melanin and PO were not incorporated into the fibres, which is a mandatory step for the formation of mature hard clots [42]. Moreover, the amount of activated PPO in plasma treated with Sc-SRP-6 was higher than in the control, likely reflecting the failure of PO and PPO to bind clot strands [21], [43]. We conclude that Sc-SRP-6 has an effect on the incorporation of melanin and PO into the plug, thus impairing the wound sealing that is essential for preventing parasite invasion [42].

The effect of Sc-SRP-6 on clotting raises the question regarding which molecules in insect plasma are being targeted. We demonstrated that Sc-SRP-6 forms a classical serpin/protease complex with a trypsin-like protein present in *G. mellonella* plasma. This trypsin was identified in cross-linked complexes in a hemolymph clot and predicted to be implicated in insect immunity as a clotting factor [44], despite not knowing the mechanism of action for this trypsin. Sc-SRP-6 also forms a covalent crosslink with two major clotting proteins, hexamerin and apolipophorin. Interestingly, these two proteins were detected among proteins in the PPO activation pathway that interact with Serpin-4 in *Manduca sexta* [45]. Hexamerin and apolipophorin have been reported to be important pro-coagulants. Hexamerin has been described to be a storage protein and a major constituent of hemolymph but could be recruited to function as a clotting factor [46]. Apolipophorin has been reported to contribute to the clotting capacity to become adhesive after LPS stimulation and interfere with the recruitment of PPO [47]. Thus, we hypothesised that Sc-SRP-6 disturbs the dynamic interaction between the clotting system and the PPO-activating cascade.

The identification and functional characterisation of Sc-SRP-6 represents a step towards the full understanding of the *S. carpocapsae* parasitic process. *S. carpocapsae* ESPs are known to contain serine proteases that facilitate host invasion and the suppression of insect defence effectors [28]; [33]; [48]; [49]. We now show that a secreted serpin can modulate the formation of hard clots, which is a part of the insect response to EPNs [50]. It is therefore logical to assume that Sc-SRP-6 is a key beneficial trait of *S. carpocapsae* as a biological control agent. This knowledge will provide new opportunities for the genetic improvement of EPNs as biological control agents and may help broaden our understanding of insect defences based on adhesive interactions leading to clotting and wound-healing.

## Supporting Information

Figure S1 **Full Sc-SRP-6 cDNA sequence.** The sequence is 1,297 bp in length including an 1,191-bp open reading frame (ORF) and a 152-bp 3′ untranslated region with a putative polyadenylation signal (AATAAA). The ORF was predicted to encode a 397-amino- acid polypeptide with a single serpin domain spanning residues 35–396 and associate with a 19-residue pre-protein and an N-terminal signal peptide spanning residues 16–17, suggesting that it is involved in a classical secretory mechanism.
(TIF)

Figure S2 **Structural model of Sc-SRP-6.** A predicted 3D model was obtained using the ITASSER server with an estimated model accuracy of 0.69±0.12 and a confidence score of −0.15. Recombinant Sc-SRP-6 showing a typical serpin fold comprising three large β-sheets (βA, B and C) and nine α-helices (hA - hI). The S4 β-strand of the βB-sheet has the RCL exposed and a predicted P1–P1′ cleavage site with Met360 (dark blue) and Ser361 (cyan) active residues.
(TIF)

Figure S3 **Activity of Sc-SRP-6 at pH 6, 7, 8 and 9.** Activity of Sc-SRP-6 was quantified at pH 6, 7, 8 and 9 using BApNA and Suc-AAPFpNA as a substrates for trypsin and chymotrypsin, respectively.
(TIF)

Table S1 **Identification of recombinant Sc-SRP-6 by MALDI-MS/MS.** A 54% sequence coverage and a significance score of 462 (P<0.05) was achieved. The confidence threshold for protein identification was set to 95% (p<0.05). Protein identification was performed using MASCOT to search the UniProtKB database (downloaded on 10/06/2012).
(DOCX)

Table S2 **Identification of Sc-SRP-6/hemolymph proteins complex by MALDI-MS/MS.** Significance scores were achieved whit confidence threshold for protein identification of 95% (p<0.05).
(DOCX)
