# Supplementary figures and images for: A Serpin Released by an Entomopathogen Impairs Clot Formation in Insect Defense System
Source: PLoS One. 2013 Jul 16;8(7):e69161. doi: 10.1371/journal.pone.0069161 (PMC3712955; doi:10.1371/journal.pone.0069161)

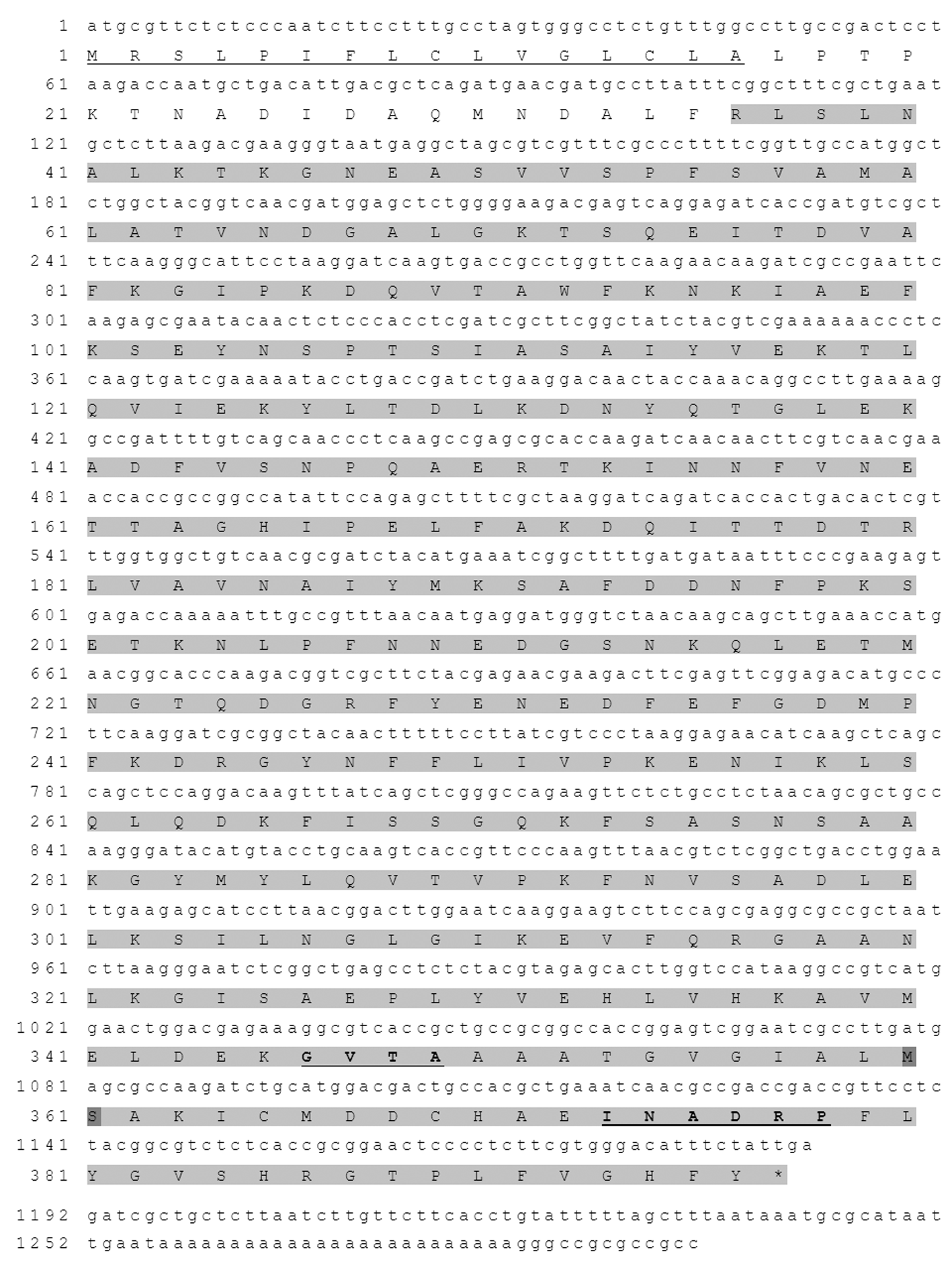

Supplement: Figure S1 — Full Sc-SRP-6 cDNA sequence. The sequence is 1,297 bp in length including an 1,191-bp open reading frame (ORF) and a 152-bp 3′ untranslated region with a putative polyadenylation signal (AATAAA). The ORF was predicted to encode a 397-amino- acid polypeptide with a single serpin domain spanning residues 35–396 and associate with a 19-residue pre-protein and an N-terminal signal peptide spanning residues 16–17, suggesting that it is involved in a classical secretory mechanism. (TIF) [file pone.0069161.s001.tif]

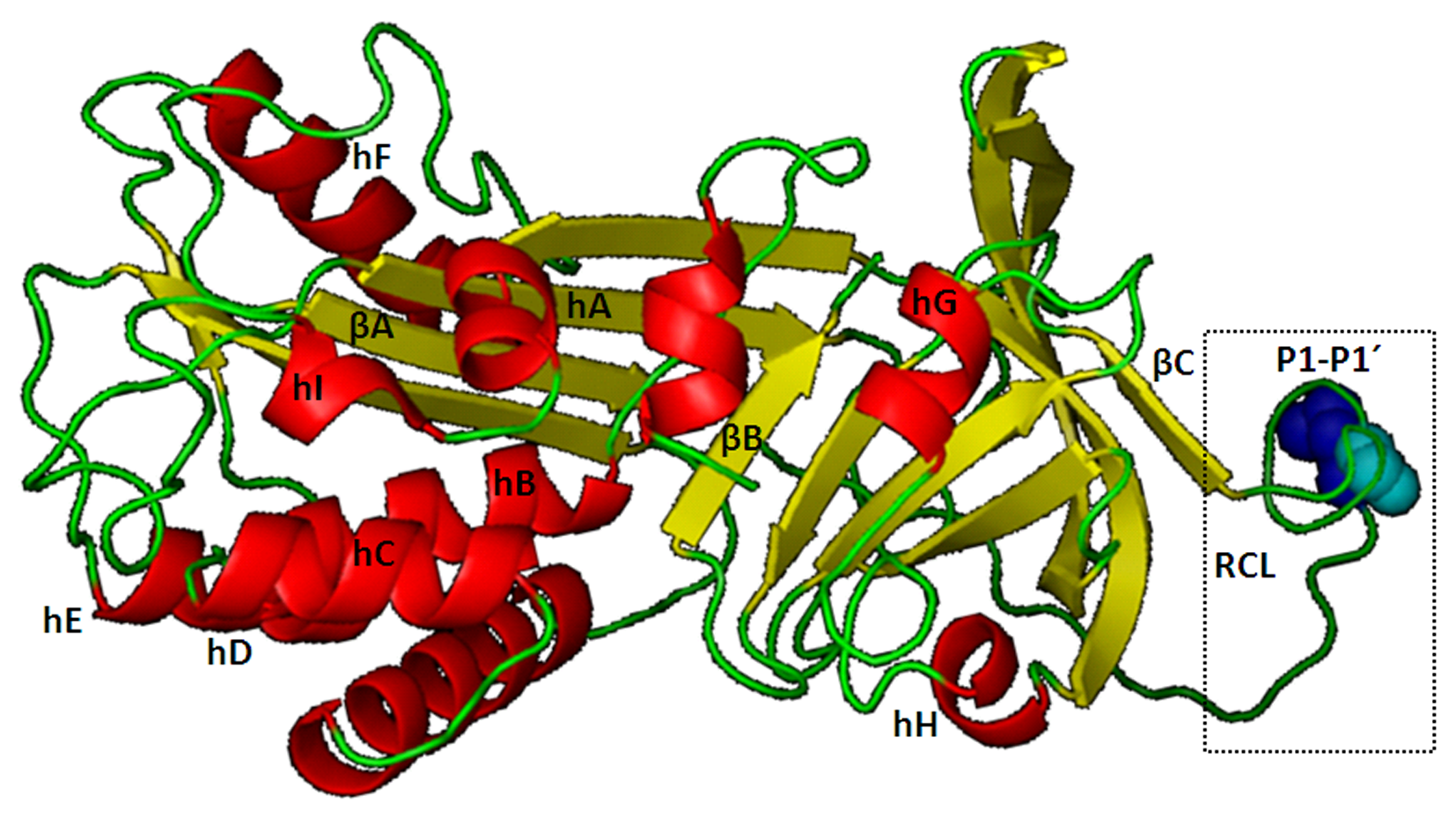

Supplement: Figure S2 — Structural model of Sc-SRP-6. A predicted 3D model was obtained using the ITASSER server with an estimated model accuracy of 0.69±0.12 and a confidence score of −0.15. Recombinant Sc-SRP-6 showing a typical serpin fold comprising three large β-sheets (βA, B and C) and nine α-helices (hA - hI). The S4 β-strand of the βB-sheet has the RCL exposed and a predicted P1–P1′ cleavage site with Met360 (dark blue) and Ser361 (cyan) active residues. (TIF) [file pone.0069161.s002.tif]

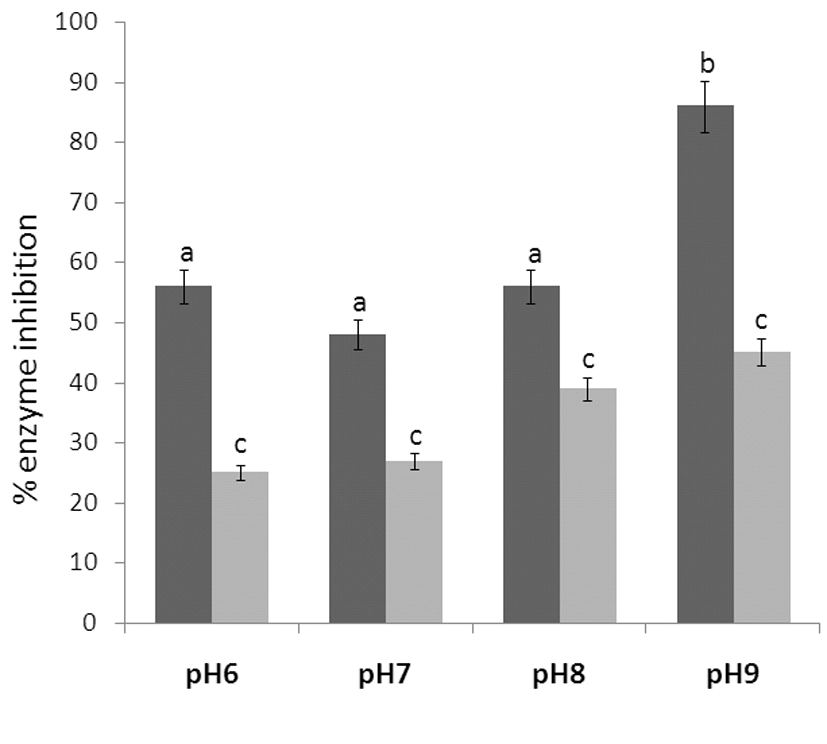

Supplement: Figure S3 — Activity of Sc-SRP-6 at pH 6, 7, 8 and 9. Activity of Sc-SRP-6 was quantified at pH 6, 7, 8 and 9 using BApNA and Suc-AAPFpNA as a substrates for trypsin and chymotrypsin, respectively. (TIF) [file pone.0069161.s003.tif]
