# Supplementary material for: A Serpin Released by an Entomopathogen Impairs Clot Formation in Insect Defense System
Source: PLoS One. 2013 Jul 16;8(7):e69161. doi: 10.1371/journal.pone.0069161 (PMC3712955; doi:10.1371/journal.pone.0069161)
Supplement: Table S1 — Identification of recombinant Sc-SRP-6 by MALDI-MS/MS. A 54% sequence coverage and a significance score of 462 (P<0.05) was achieved. The confidence threshold for protein identification was set to 95% (p<0.05). Protein identification was performed using MASCOT to search the UniProtKB database (downloaded on 10/06/2012). (DOCX) [file pone.0069161.s004.docx]

**Table S1.**

| Protein identified^(a)^ | Protein  score^(b)^ | Peptide  matches | Peptide  Calculated Mr | Peptide  error (Da) | Peptide sequence |
| --- | --- | --- | --- | --- | --- |
| gi\|306850828 | 458 | 7 | 1136.6263 | 0.0056 | LVAVNAIYMK |
|  |  |  | 1196.6594 | -0.0188 | GYNFFLIVPK |
|  |  |  | 1232.5786 | -0.0038 | ADFVSNPQAER |
|  |  |  | 1388.7452 | -0.0171 | GIPKDQVTAWFK |
|  |  |  | 1577.8413 | -0.0138 | LSQLQDKFISSGQK |
|  |  |  | 1799.8941 | -0.0179 | YLTDLKDNYQTGLEK |
|  |  |  | 2200.9048 | -0.0144 | FYENEDFEFGDMPFKDR |

1. UniProtKB database Accession number of identified protein; b) score obtained with Mowse algorithm (P< 0.05).
