# Supplementary material for: A Serpin Released by an Entomopathogen Impairs Clot Formation in Insect Defense System
Source: PLoS One. 2013 Jul 16;8(7):e69161. doi: 10.1371/journal.pone.0069161 (PMC3712955; doi:10.1371/journal.pone.0069161)
Supplement: Table S2 — Identification of Sc-SRP-6/hemolymph proteins complex by MALDI-MS/MS. Significance scores were achieved whit confidence threshold for protein identification of 95% (p<0.05). (DOCX) [file pone.0069161.s005.docx]

**Table S2**

| **Band** | **Protein identified^(a)^** | **Protein**  **score^(b)^** | **Peptide**  **Calculated Mr** | **Peptide**  **error (Da)** | **Peptide sequence** |
| --- | --- | --- | --- | --- | --- |
| **B245** | Apolipophori [Galleria mellonella]  gi\|50404098 | 380 | 1006.5  1073.6073  1101.538  1308.7522  1399.7739  1810.9869  1842.0457  2165.0386  2437.2954  2497.2112 | 0.0048  0.0123  0.0121  0.0099  0.0046  0.0058  0.0126  0.0127  0.0232  0.0261 | FDEQAQLR  GVKLSENQAK  HLTFPGNCR  FPAGITVTLPHR  QFLLAAANAITQR  GDIPDPLSLIQAYRPR  LSENQAKVEVKIVNIR  GLLGDGNNEAYDDFRLPNGK  YEELGVPESVLNAILEAHNAIR  ILQESIVEECVCNYANPFVGR |
|  | Hexamerin [Galleria mellonella]  gi\|347090 | 339 | 1031.6127  1343.7034  1659.8663  1842.0457  2230.2622  2266.1116  2648.3267 | 0.0091  0.0138  0.0066  0.0032  0.0166  0.0234  0.0263 | LAYPIWLR  RGELFFFWNK  DPAYYMIMKRVLK  LLTLFFHPHEPIHIK  QHVNENLFVNVLSVVILHR  VSSDNLLVTDEIDSASVLFNK  VGGMPFVLMVYISEYHAPNDVHR |
| **B180** | Apolipophori [Galleria mellonella]  gi\|50404098 | 387 | 1006.47  1073. 5971  1101. 5274  1308. 7452  1399.7729  1544.8992  1810.9861  1842.0437  2165.0380  2437.2853  2497.952  2897.469 | 0.0026  0.0025  0.0027  0.0029  0.0036  0.0085  0.0050  0.0106  0.0120  0.0131  0.0200  0.0835 | FDEQAQLR  GVKLSENQAK  HLTFPGNCR  FPAGITVTLPHR  QFLLAAANAITQR  KQFLLAAANAITQR  GDIPDPLSLIQAYRPR  LSENQAKVEVKIVNIR  GLLGDGNNEAYDDFRLPNGK  YEELGVPESVLNAILEAHNAIR  ILQESIVEECVCNYANPFVGR  NNYSWSTNVRTLPINICLGEQCCIR |
| **B75** | Trypsin-like [Galleria mellonella]  gi\|15072548 | 230 | 1119. 2645  1556.7875  1678.03  2001.2803  2339.8424  2435.883 | 0.0081  0.0100  0.0108  0.0110  0.0106  0.0105 | YPGVNARVSRF  HVQIWTVNQATCRN  IVVGICSWGTQCALARY  SILSAAHCFVGDPANRWRV  FVFVWLAIIAAVAAVPRNPQRI  IVVGICSWGTQCALARYPGVNARV |
| **B45** | Serpin  [Steinernema carpocapsae]  gi\|306850828 | 370 | 1136.6160  1198.4194  1239.3655  1390.6247  1486.58  1799.9008  2247.6100 | 0.0150  0.0100  0.0105  0.107  0.076  0.0112  0.085 | LVAVNAIYMK  GYNFFLIVPKE  TSQEITDVAFKG  GIPKDQVTAWFK  SAFDDNFPKSETKN  YLTDLKDNYQTGLEK  GAANLKGISAEPLYVEHLVHKA |
| **B40** | Serpin  [Steinernema carpocapsae]  gi\|306850828 | 255 | 1136.6263  1198.4249  1234.3140  1316.5000  1579.8010  1932.0848 | 0.0056  0.0050  0.0047  0.0061  0.0034  0.0069 | LVAVNAIYMK  GYNFFLIVPKE  ADFVSNPQAER  ENIKLSQLQDKF  LSQLQDKFISSGQKF  FYENEDFEFGDMPFKD |

a)UniProtKB database Accession number of identified protein; b) score obtained with Mowse algorithm (P< 0.05).
